# Direct analysis of Holliday junction resolving enzyme in a DNA origami nanostructure

**DOI:** 10.1093/nar/gku320

**Published:** 2014-05-12

**Authors:** Yuki Suzuki, Masayuki Endo, Cristina Cañas, Silvia Ayora, Juan C. Alonso, Hiroshi Sugiyama, Kunio Takeyasu

**Affiliations:** 1 Department of Chemistry, Graduate School of Science, Kyoto University, Kitashirakawa-oiwakecho, Sakyo-ku, Kyoto 606-8502, Japan; 2 CREST, Japan Science and Technology Corporation (JST), Sanbancho, Chiyoda-ku, Tokyo 102-0075, Japan; 3 Institute for Integrated Cell-Material Sciences (WPI-iCeMS), Kyoto University, Yoshida-ushinomiyacho, Sakyo-ku, Kyoto 606-8501, Japan; 4 Centro Nacional de Biotecnología, CNB-CSIC, C/Darwin 3, 28049 Madrid, Spain; 5 Laboratory of Plasma Membrane and Nuclear Signaling, Graduate School of Biostudies, Kyoto University Yoshida-konoe-cho, Sakyo-ku, Kyoto 606-8501, Japan

## Abstract

Holliday junction (HJ) resolution is a fundamental step for completion of homologous recombination. HJ resolving enzymes (resolvases) distort the junction structure upon binding and prior cleavage, raising the possibility that the reactivity of the enzyme can be affected by a particular geometry and topology at the junction. Here, we employed a DNA origami nano-scaffold in which each arm of a HJ was tethered through the base-pair hybridization, allowing us to make the junction core either flexible or inflexible by adjusting the length of the DNA arms. Both flexible and inflexible junctions bound to *Bacillus subtilis* RecU HJ resolvase, while only the flexible junction was efficiently resolved into two duplexes by this enzyme. This result indicates the importance of the structural malleability of the junction core for the reaction to proceed. Moreover, cleavage preferences of RecU-mediated reaction were addressed by analyzing morphology of the reaction products.

## INTRODUCTION

A four-way deoxyribonucleic acid (DNA) junction [Holliday junction (HJ)] is a central intermediate of homologous recombination that constitutes a ubiquitous pathway in the repair of double-stranded DNA breaks (1) and the restart of stalled replication forks (2,3). The junction is ultimately resolved into two nicked-duplex species by the action of junction-resolving enzymes (resolvases) (4,5). These enzymes exhibit high structural selectivity for HJ DNA. However, paradoxically, resolvases impose significant torsional distortion on the junctions that they recognize (6).

**Figure 1.**
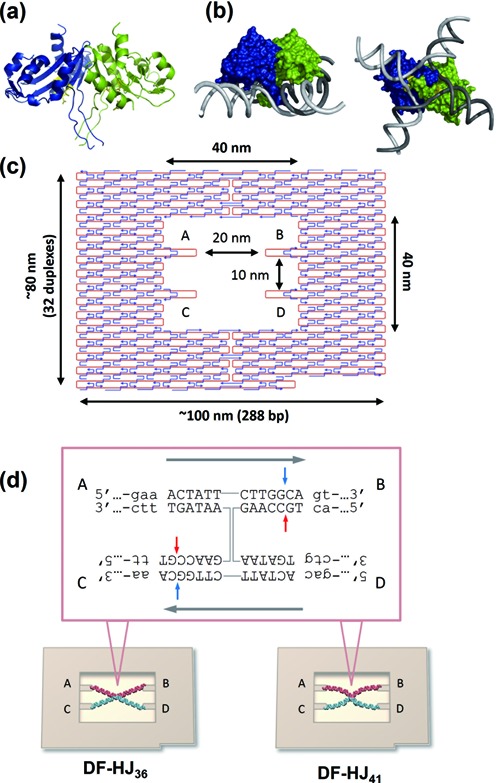
Model of a RecU dimer bound to a 4-fold symmetric HJ DNA, and scheme of the DNA-origami HJs. (**a**) Structure of *Bacillus subtilis* RecU (PDB 1ZP7). The two monomers are colored blue and green, and the active sites in yellow. The structure lacks the first 33 N-terminal residues and the end of the stalk (residues 63–79) is disordered. (**b**) A model for a RecU dimer bound to a 4-fold symmetric HJ where the phosphate backbones of the four DNA strands are shown as a tube (in gray). The figure was produced using PyMOL, and shown are a lateral and a frontal view. (**c**) Design of the DNA origami scaffold (DNA frame). (**d**) Anti-parallel HJ structures (HJ_36_ or HJ_41_) containing recognition site of RecU at their centerwere connected to the specific sites (A-B-C-D) in the DNA frame using the corresponding ssDNA sticky ends. The sequence of the core is shown. The homologous core and the non-homologous region are shown in capital letters and small letters, respectively. RecU cleavage sites are indicated by red and light blue arrows.

One of the remarkable examples is the *Bacillus subtilis* RecU resolvase (5), where the junction adopts an open structure after RecU binding (7). RecU acts as a homodimer and binds the four-way DNA junction with a dissociation constant of ∼0.5 nM (7). Although RecU cleaves on either side of the junction at a specific sequence (5′-^G^/_T_G↓C^A^/_C_-3′), recognition of and binding to the junction occur based on the structure rather than sequence preference (7). Crystallographic study of dimeric RecU revealed a mushroom-like morphology, with a cap and stalk (8,9), the stalk region of which is essential for HJ recognition (10) (Figure 1a). Biochemical studies suggest that the central hole of the HJ accommodates the stalk region, resulting two arms of the junction positioned near the active sites of the two RecU monomers (8) (Figure 1b). Thus, recognition of the junction and nicking of the DNA would be structurally connected by the distortion of the HJ. This hypothesis is supported by the characterization of RecU mutants that poorly distort the junction and fail to cleave the HJ (10). It is therefore of considerable interest to explore the role of structural malleability of the junction on the reactivity of the enzyme.

Here, we created two different framed HJ structures, flexible and inflexible, by employing a DNA origami nanoscaffold (DNA frame) described in the previous reports (11–15). The DNA frame (80 nm × 90 nm) has a vacant rectangle area (40 nm × 40 nm), in which four connection sites were introduced for hybridization of the helices of a four-way DNA junction (Figure 1c). Two antiparallel four-way junctions were introduced into the DNA frame: one with arms of 36-nucleotides (nt) and another with 41-nt arms (each assembled structure was termed as DF-HJ_36_ and DF-HJ_41_, respectively). The 36-nt junction just fitted as a tensed state in the frame, while the 41-nt junction is in a more relaxed state that can allow the flexible motion of the junction core (Figure 1d). Using these fabricated nanostructures, the effect of architectural malleability of the junction on its recognition and resolution by RecU was examined. A junction-resolving reaction was also successfully monitored using high-speed atomic force microscopy (HS-AFM).

## MATERIALS AND METHODS

### Design of DNA frame

DNA frame was designed as previously reported (11). The DNA sequence design of the DNA frame structure followed the rules of the DNA origami method. The sequence of the M13mp18 was used, and the staple strands (most of them are 32-nt) were assigned for the formation of the designed DNA frame. The DNA frame lacks the right bottom corner for the identification of the orientation of the frame (orientation marker). The sequences and positions of the staple strands are listed in the supporting information of ref (11).

### DNA frame formation and Integration of the Holliday Junction structures

The DNA frame and the Holliday Junction structure were assembled in a 25 μl solution containing 10 nM M13mp18 single-stranded DNA (New England Biolabs), 50 nM staple strands (226 strands), 20 mM Tris–HCl (pH 7.6), 1 mM EDTA, and 10 mM MgCl_2_. The mixture was annealed from 85 to 15°C at a rate of −1.0°C/min. The four-way junctions, which contain a 13-bp homologous core, were constructed by annealing four strands HJ_36_-a, HJ_36_-b, HJ_36_-c and HJ_36_-d (for 36-nt junction) or HJ_41_-a, HJ_41_-b, HJ_41_-c and HJ_41_-d (for 41-nt junction). The sequences of the oligonucleotides used for constructing four-way junctions are seen in Supplementary Figure S1. The HJ structures were subsequently incorporated into the DNA frame by annealing the mixture from 40 to 15°C at a rate of −0.5°C/min using thermal cycler. The sample was purified by gel-filtration chromatography (GE sephacryl-400, GE Healthcare Japan, Tokyo, Japan) to remove excess amounts of the staple strands and the HJ structures (Supplementary Figure S2).

### RecU protein purification

*Escherichia. coli* BL21(DE3) [pLysS] cells containing pCB210-borne *rec*U gene (7) were resuspended in buffer A (50 mM Tris–HCl, pH 7.5/1 mM DTT/5% glycerol) containing 100 mM NaCl and lysed with a French press. RecU was purified as previously described (7) to >99% purity, as judged by 15% sodium dodecyl sulfate-polyacrylamide gel electrophoresis and matrix-assisted laser desorption ionization time-of-flight analysis. The RecU concentration was determined by using a 24 900 M^−1^ cm^−1^ molar extinction coefficient and is expressed as mol of protein monomers.

### AFM analysis of RecU binding and RecU-mediated HJ resolution

Binding reactions were performed by mixing 30 nM of RecU and 10 nM of the DNA frame carrying 36-nt junction or 41-nt junction in a Tris-buffer A [20 mM Tris–HCl (pH 7.6), 1 mM EDTA, 50 mM NaCl and 3 mM MgCl_2_] for 30 min at 30°C. Cleavage reactions were performed by mixing 30 nM of RecU and 10 nM of the DNA frame carrying 36-nt junction or 41-nt junction in a Tris-buffer B [20 mM Tris–HCl (pH 7.6), 1 mM EDTA, 50 mM NaCl and 10 mM MgCl_2_] for 30 min at 30°C. For AFM imaging, 2 μl of sample was deposited onto a freshly cleaved mica disc immediately after the above described incubation. After 1 min, the sample was rinsed with 10 μl of the Tris-buffer A (for binding assay) or B (for cleavage assay) and imaged in the same buffer. The appearance frequency of each distinct structure was calculated from the obtained images. The broken structures were not taken into account for the yield calculations.

### Time-lapse HS-AFM imaging of individual reactions

The resolution reactions were imaged as follows: 30 nM of RecU was pre-incubated with 10 nM of the DNA frame carrying 41-nt junction at 30°C for 5 min in a Tris-buffer A. After the incubation, 2 μl of the sample was immediately deposited onto a freshly cleaved mica disc. After 30 s incubation, the sample was rinsed with 10 μl of the Tris-buffer B and imaged in the same buffer.

### AFM imaging

The imaging was performed using prototype high-speed AFMs (Olympus Corporation, Tokyo, Japan) (16,17). The sample was imaged in the imaging buffer solution at ambient temperature using small cantilevers with dimensions (L × W × H) of 10 μm × 2 μm × 0.1 μm (Olympus Corporation, Tokyo, Japan). These cantilevers have a spring constant of 0.1–0.2 N/m with a resonant frequency of 400–1000 kHz in water. A sharp probe was deposited on each cantilevers using electron beam deposition by Nanotools (Munich, Germany). The 320 × 240 pixels images were obtained at a scan rate of 0.2–0.5 frame/s.

## RESULTS AND DISCUSSIONS

### Preparation and analysis of the framed Holliday Junctions

**Figure 2.**
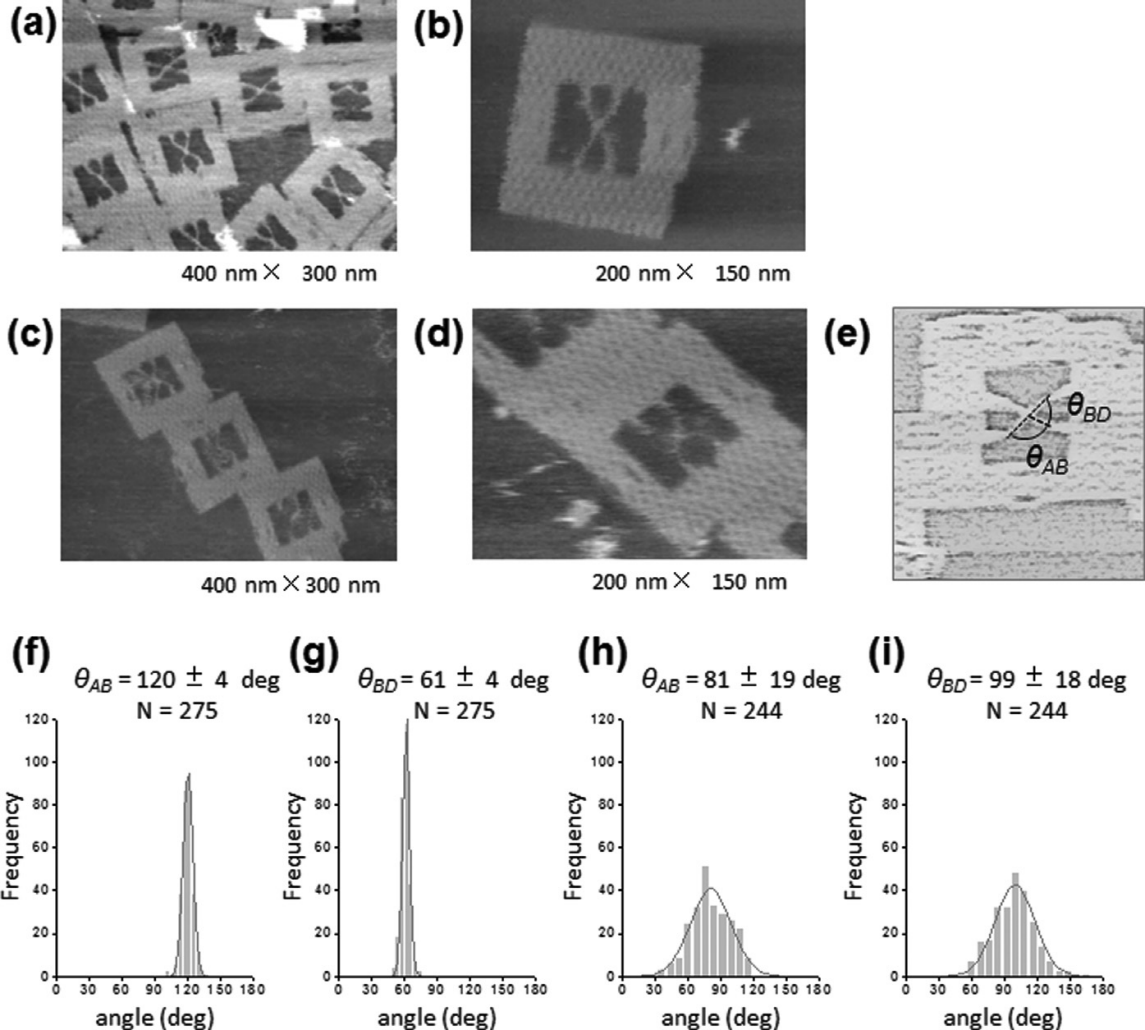
AFM analyses of HJ structures incorporated in the DNA frame. (**a**, **b**) AFM images of DF-HJ_36_. (**c**, **d**) AFM images of DF-HJ_41_. Image size: (a, c) 400 nm × 300 nm; (b, d) 200 nm × 150 nm. The DNA frames were frequently observed to be connected each other by π-stacking interactions. (**e**) Scheme illustrating the procedure for measurement of the angles between DNA arms. (**f**, **g**) Histograms of the angle *θ*_AB_ (the angle between DNA arm A and B) and the angle *θ*_BD_ (the angle between DNA arms B and D) for DF-HJ_36_. (**h**, **i**) Histograms of the angle *θ*_AB_ and *θ*_BD_ for DF-HJ_41_.

**Figure 3.**
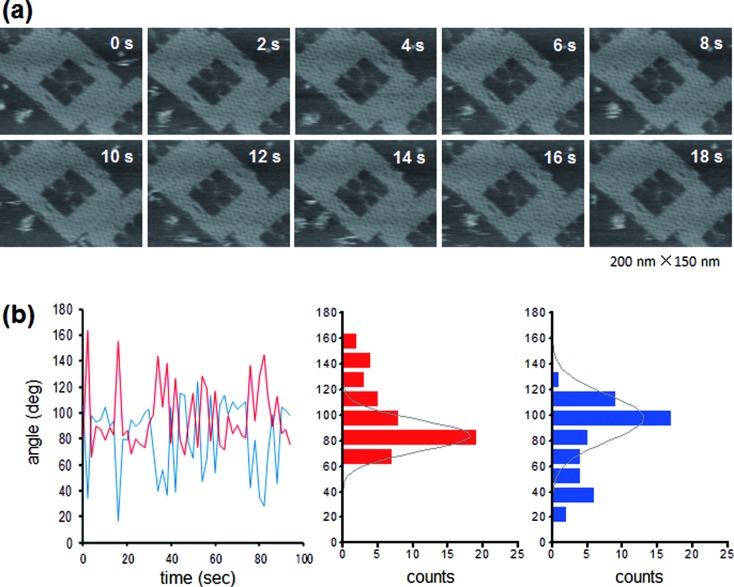
Fluctuating motion of the junction core of DF-HJ_41_. (**a**) Successive HS-AFM images of DF-HJ_41_ obtained at 0.5 frame/s. The elapsed time is shown in each image. Image size: 200 nm × 150 nm. For the complete movie, see Supplementary Movie S1. (**b**) For all 48 images, collected in a 2 s interval, the angles *θ*_AB_ (red) and *θ*_BD_ (blue) were measured and are shown in the trace, along with the histograms.

AFM images of the assembled structures revealed a clear X-shaped junction in the DNA frame (Figure 2a–d). The flexibility of the junction core was evaluated by measuring the angle between arms (Figure 2e). In the case of the 36-nt junction, frequency distribution of the angle *θ*_AB_ and angle *θ*_BD_ between arms showed a sharp peak at 120 ± 4° and 61 ± 4°, respectively (Figure 2f and g). These values agreed well with expected angles when the junction is attached to the frame. In the case of the 41-nt junction, the frequency distribution of these angles showed a much broader peak at 81 ± 19° and 99 ± 18° (Figure 2h and i). Note that the values of standard deviation were about 5-times larger than those for the 36-nt junction, allowing us to assume that the 41-nt junction in the DNA frame was flexible enough to change the junction structure, unlike the 36-nt junction. Time-lapse AFM imaging of DF-HJ_41_ also showed this flexible feature (Figure 3a, See also Supplementary Movie S1). It is noted that the DNA frame was stably attached on the mica surface, while an inside HJ was mobile enough on the surface. Figure 3b shows the variation with time of *θ*_AB_ and *θ*_BD_ of the DF-HJ_41_, together with histograms of the measured angles. The histograms provided mean values of *θ*_AB_ = 84 ± 12° and *θ*_BD_ = 98 ± 18° which are similar to the distribution in Figure 2h and i, showing the flexible motion of each arm with the extent of ±12–18° around the junction core. This type of flexible motion of the framed HJ was restricted when its arms were shortened to 36-nt (Supplementary Figure S3).

### Binding of RecU to the framed Holliday Junctions

**Figure 4.**
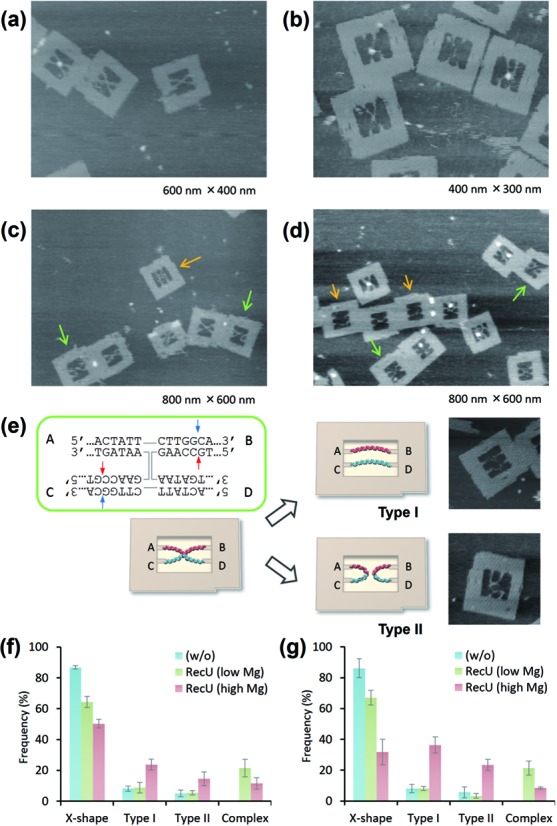
RecU-HJ complex formation and junction resolution in the DNA frame. Representative AFM images of (**a**) DF-HJ_36_ and (**b**) DF-HJ_41_ after incubation with RecU at low Mg^2+^ concentrations. Representative AFM images of (**c**) DF-HJ_36_ and (**d**) DF-HJ_41_ after the reaction with RecU at high Mg^2+^ concentrations. Colored arrows in the images highlight the Type I (orange) and Type II (green) products. Image size: 800 nm × 600 nm. (**e**) Schematic of possible configurations of the resolved strands in the DF. Quantification of the junction-resolving reaction by RecU for (**f**) DF-HJ_36_ and (**g**) for DF-HJ_41_. Appearance of X-shape, Type I, Type II and RecU bound complex were counted. The data were collected from three independent measurements.

The binding of RecU to these HJs incorporated into the DNA frame was examined by AFM. RecU requires high concentrations of Mg^2+^ (10–15 mM) to resolve the junction (7). Therefore, the binding reaction was performed in the presence of a lower concentration of MgCl_2_ (3 mM) to avoid the cleavage. Assembled DF-HJs were incubated with RecU at 30°C for 30 min, and then the mixture was imaged (Figure 4a and b). The height of the RecU bound junction was about 4.9 nm, which was ∼3 nm higher than that of the center of the junction in the absence of protein (Supplementary Figure S4). The enzyme was seen to specifically recognize and bind to the four-way junction. The binding efficiency of the 36-nt junction and the 41-nt junction under this condition showed a similar value (∼21%) (Supplementary Table S1). Interestingly, even though HJ was tense and inflexible in the DF-HJ_36_, 21% of them were bound with RecU, implying the capability of RecU of distorting this junction to allow its binding.

### Reactivity of RecU with flexible/inflexible Holliday Junctions

**Figure 5.**
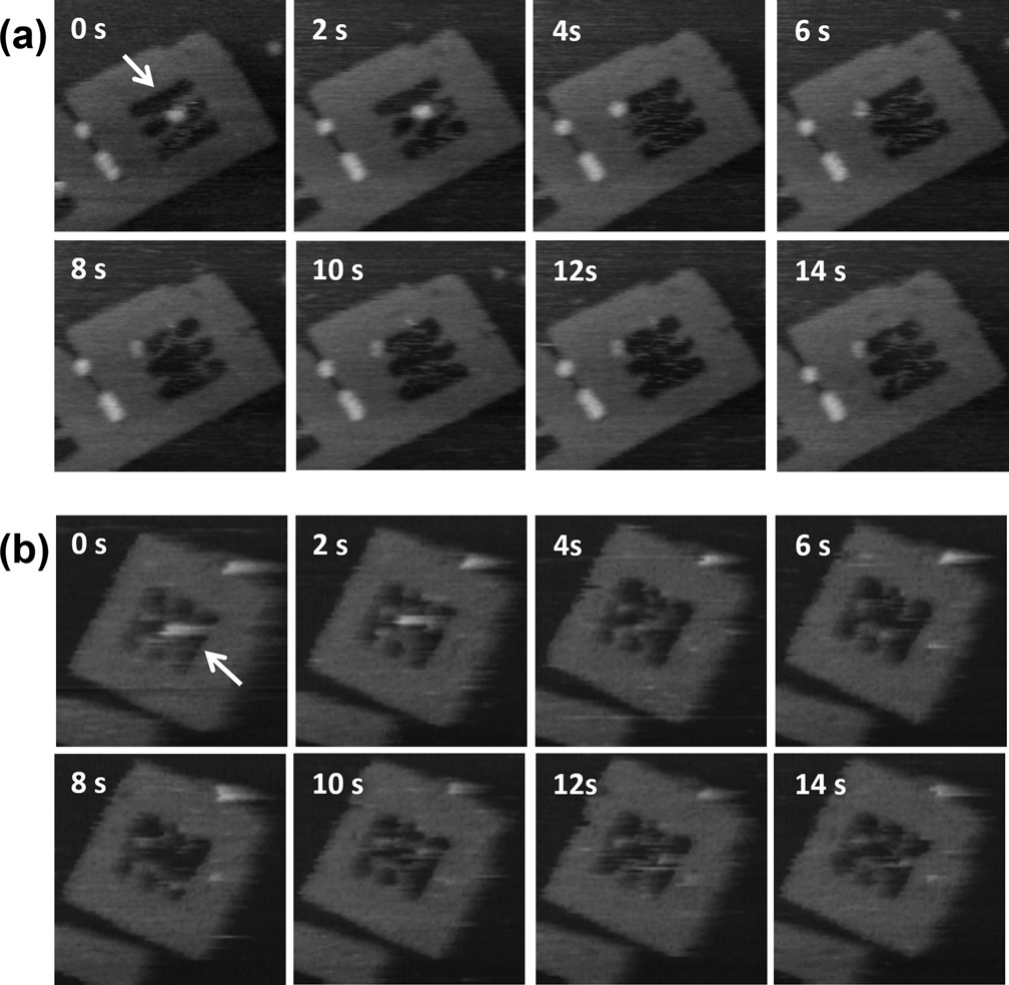
Junction resolution by RecU. (**a**) Time-lapse images of the resolution of a selected DF-HJ_41_–RecU complex (white arrow) which resulted in two separated strands (type I). (**b**) Time-lapse images of the resolution of a DF-HJ_41_–RecU complex (white arrow) which produced a double-loop structure (type II). Images were obtained at scan rate of 0.5 frame/s. The elapsed time is shown in each image. Image size: 200 nm × 200 nm.

To examine whether differences in core flexibility affect the reaction efficiency, each DF-HJ was incubated with RecU in the presence of 10 mM of MgCl_2_ for 30 min, and imaged in the same buffer (Figure 4c and d). Note that both junctions contain the same 13-bp homologous junction core, which carries two pairs of symmetric cleavage sites (Figure 4e, blue and red arrows). Therefore, it is expected that the nicking reaction will result in two possible structural patterns: two separate parallel DNA strands (Type I) or two separate double-looped DNA structures (Type II) (Figure 4e and Supplementary Figure S5). In the case of DF-HJ_36_, incubation with RecU resulted in ∼39% of the HJ resolved (Type I: 24%, Type II: 15%), with the protein-unbound DNA frames retaining their X-shape structure (50%) (Figure 4f and Supplementary Table S1). On the other hand, when RecU was incubated with DF-HJ_41_, 60% of the HJ was resolved (Type I: 36%, Type II: 24%) and only 30% retained the X-shape structure (Figure 4g and Supplementary Table S1). HS-AFM imaging of the junction-resolving reaction in the individual DF-HJ_41_ directly provided the evidence for both reaction patterns. The sample was pre-incubated with RecU in the buffer containing 3 mM MgCl_2_ and then imaged in the buffer containing 10 mM of MgCl_2_. Figure 5 shows the snapshot images of the reaction (see also Supplementary Movie S2). At the initial frame of Figure 5a, RecU protein was imaged bound to the center of the junction core, which is located at around 40-bp from each connection point. The protein dissociated at 4 s, and two separate parallel double stranded DNAs were observed in the DNA frame. The other type of the reaction, production of the double-loop was also successfully visualized (Figure 5b, see also Supplementary Movie S3). The complex was seen until 2 s. At 4 s, the protein was seen to dissociate leaving double-looped structure in the DNA frame. These results show that the 41-nt HJ is malleable enough to allow not only the binding but also the nicking reaction, even though DNA frames were placed on the mica surface. It is noteworthy that type I configurations (due to cleavage on the exchanging strands) were more frequently observed as reaction products in our DNA frame system (Figure 4f, g and Supplementary Table S1). Previous *in vitro* studies showed that RecU has no apparent preferred orientation for cleavage (7). However, on the other hand, RecU resolves HJs with non-crossover preference *in vivo* (18) by symmetrically cleaving continuous strands (19). Our results obtained with framed HJs strongly suggest that DNA topology determines the *in situ* outcome of the reaction.

The results obtained here also show that the DF-HJ_36_ was not as effectively resolved as the DF-HJ_41_, and the relaxed junction is a better substrate for RecU than the tensed HJ structure, although the sequence of the junction core was the same in both the DF-HJ_36_ and DF-HJ_41_ junctions. Considering that both structures can be bound by RecU, the reactivity seems to be affected by whether the junction can accommodate the subsequent conformational changes which are required for the DNA cleavage. The tensed junction in DF-HJ_36_ might be less capable of adopting this requisite changes, resulting in the poor resolution of the junction. In contrast, the relaxed four-way junction in DF-HJ_41_ is malleable enough to allow RecU to complete the reaction. A four-way junction is a structurally polymorphic system that can adopt various conformers (20–22). Structural dynamics of the four-way junctions have been investigated by both biochemical (20,23) and single molecule analyses (22,24). However, there has been incomplete information on how those dynamic features of the HJ contribute to the reactivity of resolvases. DNA origami-based single-molecule approaches illustrated here could provide previously unattainable clues to the interaction/working mechanisms of junction binding enzymes in the context of HJ topology and flexibility.

## CONCLUSION

In conclusion, we here report the direct single-molecule analysis of the HJ-resolving reaction mediated by RecU. The flexibility of the substrate HJ was regulated in the defined nanoscaffold, a DNA frame, which was constructed by the DNA origami method. This method allowed us to examine the role of junction malleability in the reaction. The results obtained demonstrated the strong capability of RecU to distort the junction and the importance of junction malleability especially in the nicking reaction of the junction. The presented method should be applicable to investigate the mechanisms used by other junction binding enzymes which are involved in branch migration and junction resolving reaction.

## SUPPLEMENTARY DATA

Supplementary Data are available at NAR Online.

SUPPLEMENTARY DATA
