# Supplementary material for: Direct analysis of Holliday junction resolving enzyme in a DNA origami nanostructure
Source: Nucleic Acids Res. 2014 May 12;42(11):7421–8. doi: 10.1093/nar/gku320 (PMC4066755; doi:10.1093/nar/gku320)
Supplement: SUPPLEMENTARY DATA [file supp_42_11_7421__index.html]

Direct analysis of Holliday junction resolving enzyme in a DNA origami nanostructure — Direct analysis of Holliday junction resolving enzyme in a DNA origami nanostructure — Direct analysis of Holliday junction resolving enzyme in a DNA origami nanostructure — SUPPLEMENTARY DATA 

# Direct analysis of Holliday junction resolving enzyme in a DNA origami nanostructure

## SUPPLEMENTARY DATA

**Files in this Data Supplement:**

- SUPPLEMENTARY DATA
- SUPPLEMENTARY DATA
