# Supplementary material for: Direct analysis of Holliday junction resolving enzyme in a DNA origami nanostructure
Source: Nucleic Acids Res. 2014 May 12;42(11):7421–8. doi: 10.1093/nar/gku320 (PMC4066755; doi:10.1093/nar/gku320)
Supplement: SUPPLEMENTARY DATA [file supp_gku320_nar-00191-f-2014-File007.pdf]

Supplementary Information

# Direct analysis of Holliday junction resolving enzyme in a DNA origami nanostructure

Yuki Suzuki[1,5], Masayuki Endo[2,5], Cristina Cañas[3], Silvia Ayora[3], Juan C. Alonso[3], Hiroshi Sugiyama[1,2,5], and Kunio Takeyasu[4*]

[1]Department of Chemistry, Graduate School of Science, Kyoto University, Kitashirakawa-oiwakecho, Sakyo-ku, Kyoto 606-8502, Japan, [2]Institute for Integrated Cell-Material Sciences (WPI-iCeMS), Kyoto University, Yoshida-ushinomiyacho, Sakyo-ku, Kyoto, 606-8501, Japan, [3]Centro Nacional de Biotecnología, CNB-CSIC, C/Darwin 3, 28049 Madrid, Spain, [4]Laboratory of Plasma Membrane and Nuclear Signaling, Graduate School of Biostudies, Kyoto University Yoshida-konoe-cho, Sakyo-ku, Kyoto 606-8501, Japan, [5]CREST, Japan Science and Technology Corporation (JST), Sanbancho, Chiyoda-ku, Tokyo 102-0075, Japan

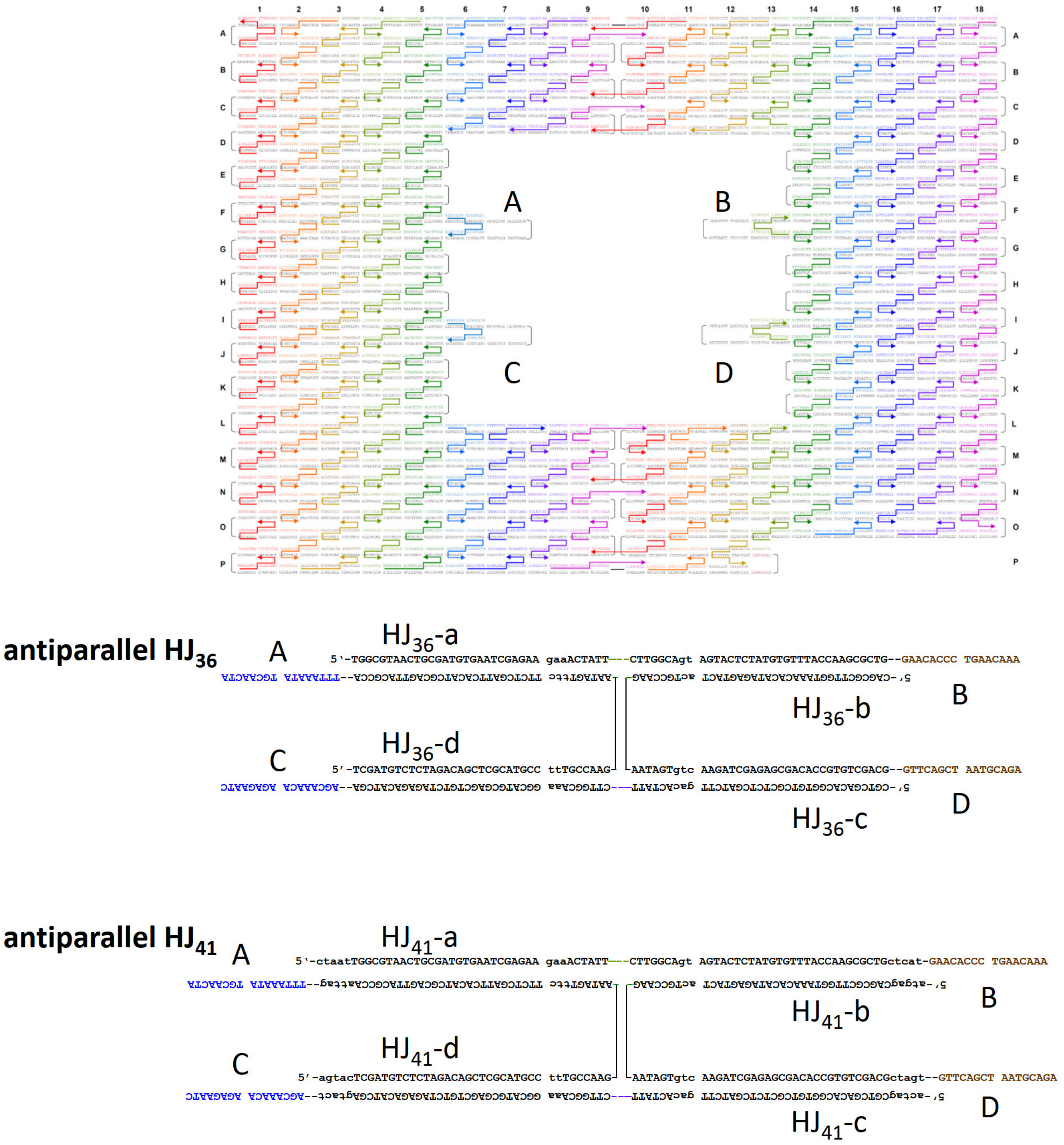


**Figure S1.** Holliday Junctions $HJ_{36}$ and $HJ_{41}$ in the antiparallel arrangement for incorporation to DNA frame. The four-way junctions were constructed by annealing four strands $HJ_{36}$-a, $HJ_{36}$-b, $HJ_{36}$-c, and $HJ_{36}$-d (for 36-nt junction) or $HJ_{41}$-a, $HJ_{41}$-b, $HJ_{41}$-c, and $HJ_{41}$-d (for 41-nt junction). The upper case letters indicate four different connection sites.

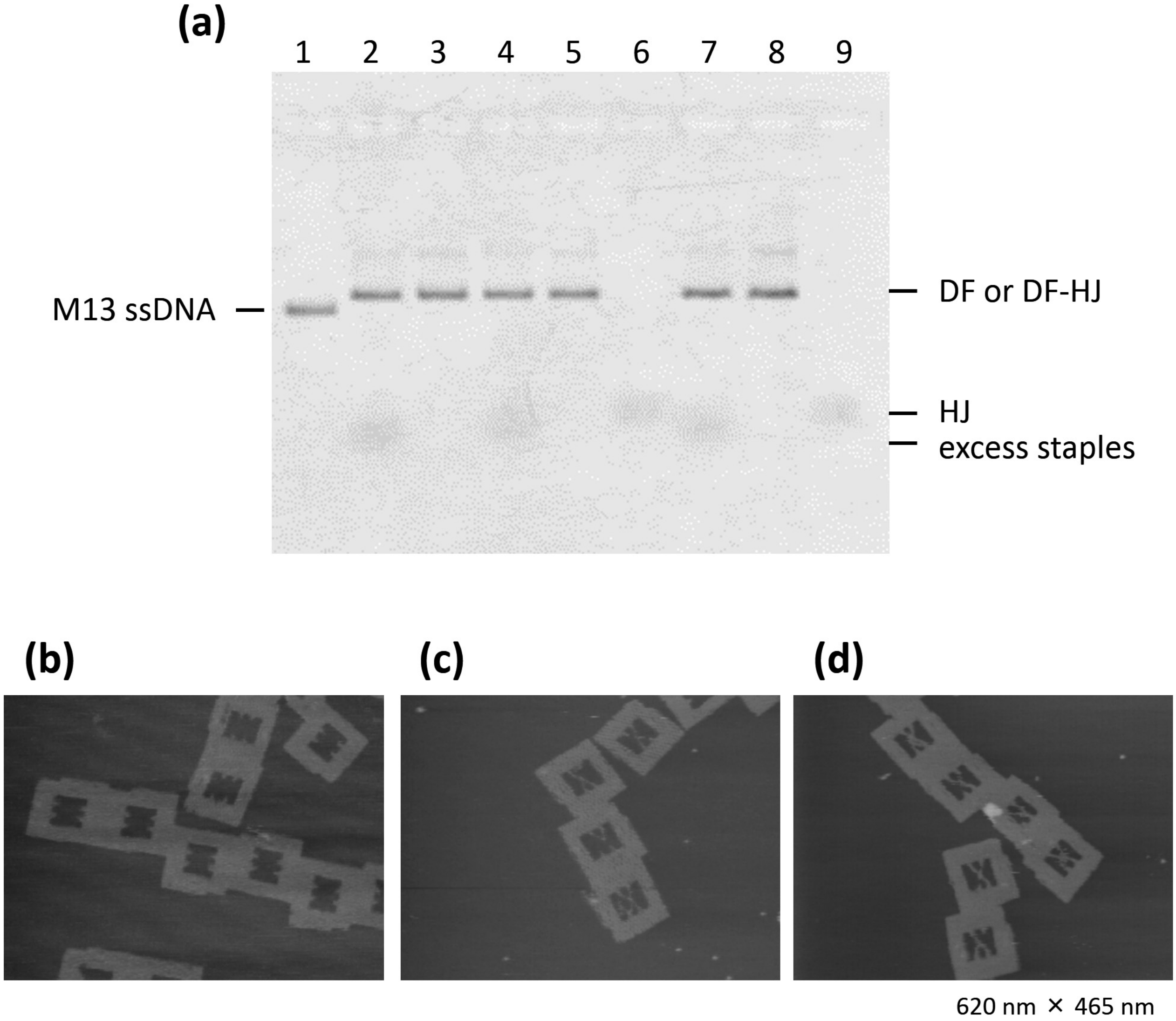


**Figure S2**. (a) Agarose gel (0.7%) of the assembled structures. Lane 1: M13ssDNA scaffold. Lane2: folded DNA frame (DF). Lane 3: purified DF. Lane 4: folded DF-$HJ_{36}$. Lane 5: purified DF-$HJ_{36}$. Lane 6: $HJ_{36}$. Lane 7: folded DF-$HJ_{41}$. Lane 8: purified DF-$HJ_{41}$. Lane 9: $HJ_{41}$. Upper bands in Lane 2-5, 7, and 8 reflect $\pi$-stacked DNA frames. The samples were analyzed using a 0.7% agarose gel with 10 mM $MgCl_2$ in a TBE (Tris-borate-EDTA) buffer under 90 V at 4 °C. After electrophoresis, the gel was stained with ethidium bromide. (b-d) AFM images of the purified structures. (b) purified DF, (c) purified DF-$HJ_{36}$ and (d) purified DF-$HJ_{41}$. Image size: 620 nm × 415 nm.

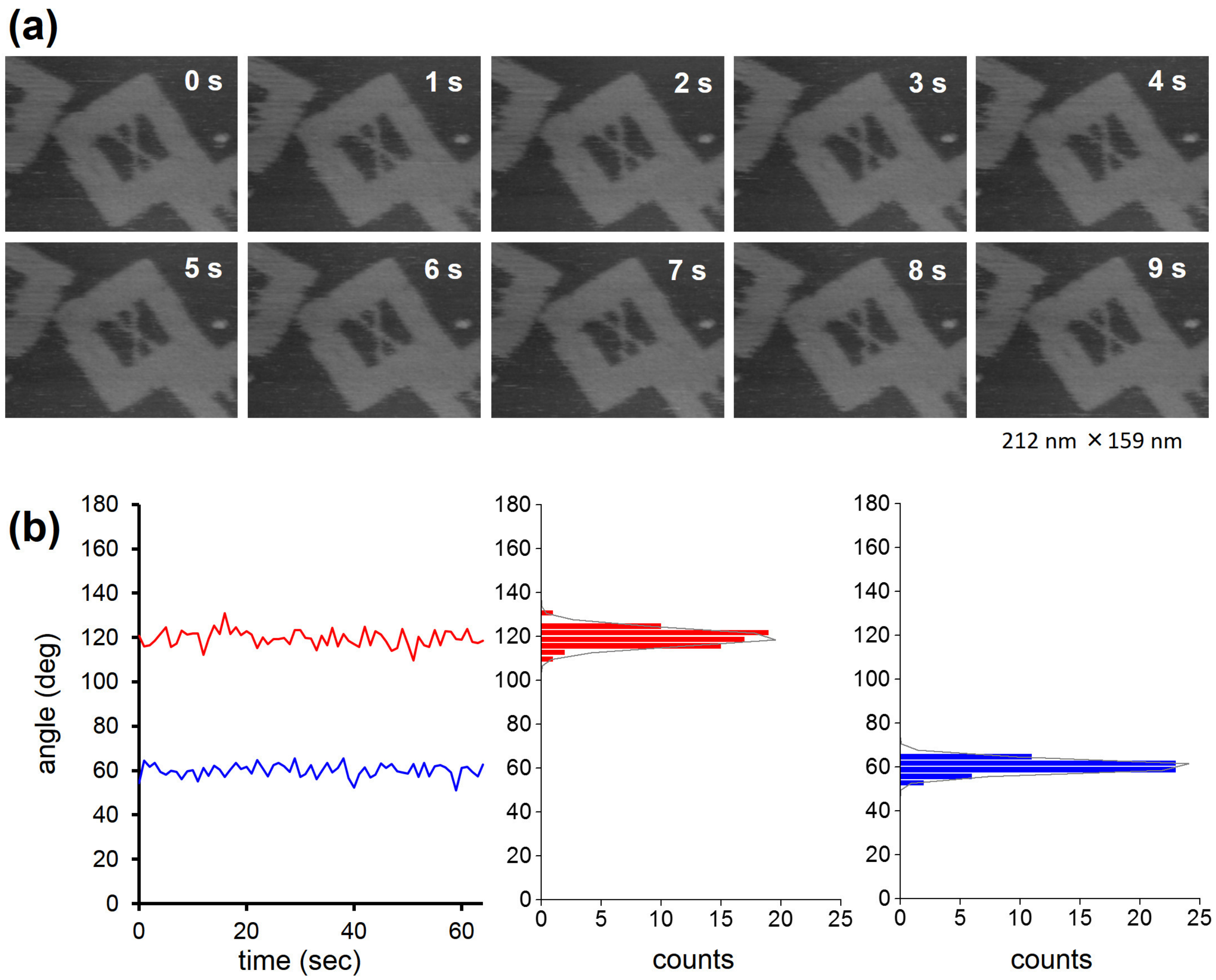


**Figure S3.** Restricted flexibility of the junction core of DF-$HJ_{36}$. (a) Successive HS-AFM images of DF-$HJ_{36}$ obtained at 1.0 frame per second. The elapsed time is shown in each image. Image size: 212 nm × 159 nm. (b) For all 65 images, collected in a 1 s interval, the angles $\theta_{AB}$ (red) and $\theta_{BD}$ (blue) were measured and are shown in the trace, along with the histograms. The histogram provided mean values of $\theta_{AB}$ = 120 ± 4 deg and $\theta_{BD}$ = 60 ± 3 deg. For the complete movie, see Supplementary Movie S4.

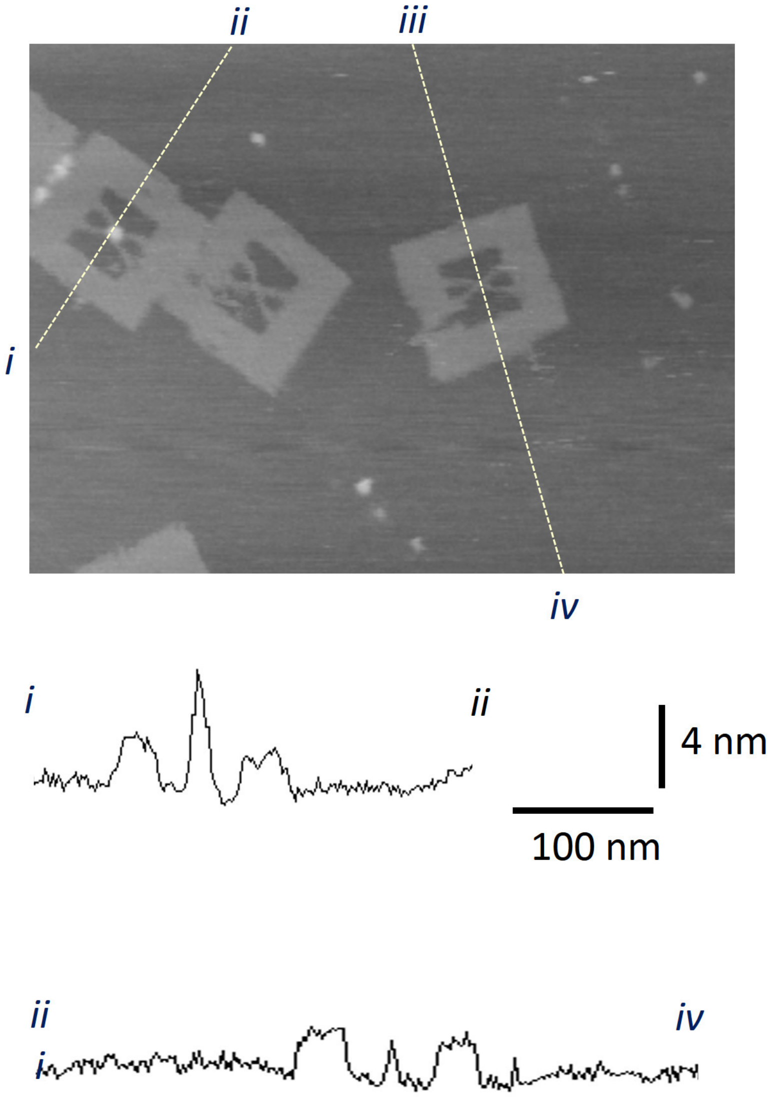


**Figure S4.** Section profiles at the positions indicated by the lines in the AFM image. *i-ii*: Section through a RecU-HJ complex. *iii-iv*: Section through a HJ center.

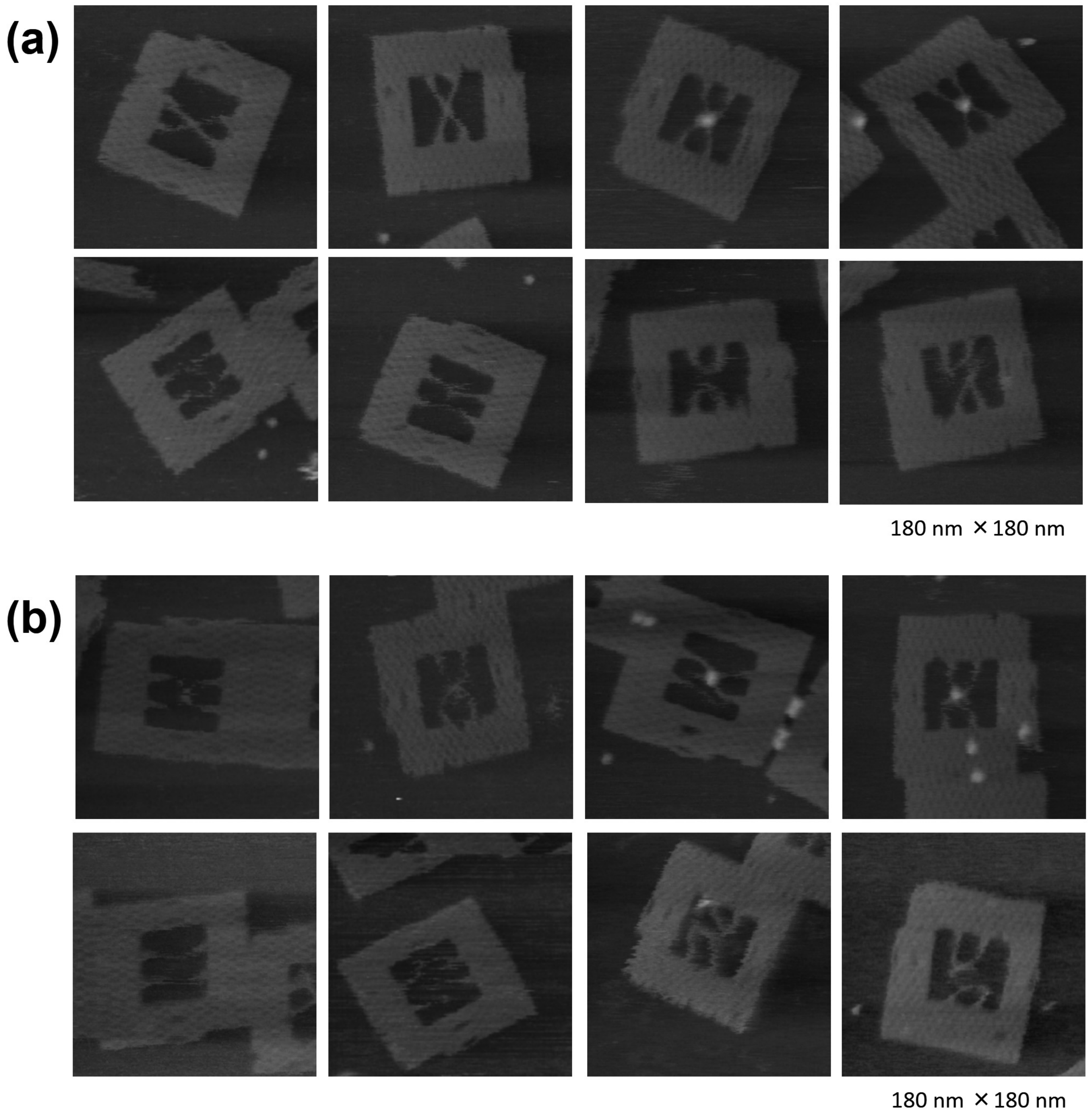


**Figure S5.** Gallery of the four types of structures observed after incubation with RecU (two X-shapes, upper left; two synaptic complexes, upper right; two separate parallel DNA strands, bottom left; two separate double looped DNA structures, bottom right) derived from (a) DF-$HJ_{36}$ and from (b) DF-$HJ_{41}$. Image size: 180 nm × 180 nm.

**Table S1.** Population of X-shape, Type I product (two separate parallel DNA strands), Type II product (two separate double looped DNA structures) and RecU bound complex.

**DF-HJ$_{36}$**

| | X-shape | Type I | Type II | Complex | Total number |
|---|---|---|---|---|---|
| w/o RecU | 86.8 ± 1.2 % | 8.2 ± 1.6 % | 5.0 ± 2.2 % | | 275 |
| + RecU 3 mM $Mg^{2+}$ | 64.3 ± 3.7 % | 8.8 ± 3.4 % | 5.4 ± 1.4 % | 21.5 ± 5.7 % | 257 |
| + RecU 10 mM $Mg^{2+}$ | 50.2 ± 2.8 % | 23.7 ± 3.5 % | 14.5 ± 4.5 % | 11.5 ± 3.8 % | 257 |

**DF-HJ$_{41}$**

| | X-shape | Type I | Type II | Complex | Total number |
|---|---|---|---|---|---|
| w/o RecU | 86.2 ± 6.2 % | 8.2 ± 2.7 % | 5.6 ± 3.6 % | | 244 |
| + RecU 3 mM $Mg^{2+}$ | 67.1 ± 4.7 % | 8.2 ± 1.3 % | 3.3 ± 1.5 % | 21.4 ± 4.6 % | 213 |
| + RecU 10 mM $Mg^{2+}$ | 31.8 ± 8.2 % | 36.3 ± 5.2 % | 23.4 ± 3.7 % | 8.5 ± 0.6 % | 201 |

All the measurements were repeated three times. The values are represented as mean ± SD.

**Movie S1.** HS-AFM images of a DF-$HJ_{41}$. Scanning rate: 0.5 frame per second, Image size: 200 nm × 150 nm. Movie is played two times faster.

**Movie S2.** HS-AFM images of a DF-$HJ_{41}$ complex which produced two separate parallel DNA strands (type I product). Scanning rate: 0.5 frame per second, Image size: 200 nm × 200 nm. Movie is played two times faster.

**Movie S3.** HS-AFM images of a DF-$HJ_{41}$ complex which produced two separate double looped DNA structures (type II product). Scanning rate: 0.5 frame per second, Image size: 200 nm × 200 nm. Movie is played two times faster.

**Movie S4**. HS-AFM images of a DF-$HJ_{36}$. Scanning rate: 1.0 frame per second, Image size: 212 nm × 159 nm.
